# Severity of Locomotive Syndrome Increases the Risk of Mortality: A Retrospective Study of Patients With Osteoporosis

**DOI:** 10.7759/cureus.83635

**Published:** 2025-05-07

**Authors:** Ryoma Asahi, Yutaka Nakamura, Masayoshi Kanai, Kohei Maruya, Satoshi Asano

**Affiliations:** 1 Department of Physical Therapy, School of Health Sciences, Japan University of Health Sciences, Satte, JPN; 2 Saitama Spine Center, Higashi Saitama General Hospital, Satte, JPN; 3 School of Health Sciences, Japan University of Health Sciences, Satte, JPN

**Keywords:** incidence, locomotive syndrome, mortality, osteoporosis, outpatients, severity of locomotive syndrome

## Abstract

Purpose: In female patients with osteoporosis, low body mass index (BMI), impaired locomotive function, and low bone mineral density (BMD) are presumed to be associated with an increased risk of mortality; however, the specific factors contributing to all-cause mortality have not yet been clearly identified. The purpose of this study was to determine the incidence of death among osteoporotic women in the community and to explore the factors involved in mortality risk.

Methods: A total of 451 postmenopausal women undergoing outpatient osteoporosis treatment at our hospital between November 2013 and March 2024 were longitudinally evaluated in this study. Underlying diseases, history of femoral and vertebral fractures, femoral and lumbar spine BMD, locomotive syndrome (LS) risk tests, and BMI were obtained at baseline. The LS risk test results were utilized to classify LS severity, which was assessed on a four-point scale ranging from stage 0 (robust) to stage 3 (severe deterioration). This study used all-cause mortality as the outcome, and we examined factors related to mortality using Cox proportional hazards regression analysis. Covariates included in the Cox proportional hazards regression analysis were age, BMI, history of femoral and vertebral fractures, lumbar spine and femoral BMD, underlying diseases, and LS severity.

Results: The mean follow-up was 5.0 years (2.7 years), and death occurred in 20 of 451 patients. The incidence of mortality in this study was 0.91 cases per 100 person-years of risk. Cox proportional hazards regression analysis adjusted for all variables, and this study showed that cardiovascular disease (CD) (hazard ratio (HR) = 5.209, 95% confidence interval (CI) 1.794-15.128, p-value = 0.002) and severity of LS (HR = 2.006, 95% CI 1.063-3.784, 0.032) were shown to be independent risk factors for the occurrence of all-cause mortality.

Conclusion: To the best of our knowledge, this is the first study to demonstrate a significant association between the severity of LS and mortality in patients with osteoporosis. The severity of LS may be useful as a screening tool for all-cause mortality in women with osteoporosis.

## Introduction

Osteoporosis is on the rise worldwide as an important public health and societal issue [1, 2]. It is estimated that 15.9 million people in Japan are affected by osteoporosis, and the number of patients with fractures increases every year due to the aging population, especially among females [3, 4]. Fractures, particularly femoral and vertebral ones, have been reported to have a higher mortality risk [5-7].

Osteoporotic patients are more likely to be female. In women, estrogen levels tend to decline with the onset of menopause. Estrogen plays a key role in maintaining bone mass by shortening the lifespan of osteoclasts and enhancing the survival of osteoblasts and osteocytes [8]. A previous study showed significantly lower body mass index (BMI), bone mineral density (BMD), and motor functions compared to women without osteoporosis [9, 10]. Suzuki et al. showed that low BMD status independently increases the risk of mortality in older females, and osteoporotic patients have a significantly higher risk of mortality than those without osteoporosis [11]. The mortality risk should be the focus in both fractures and low BMD in osteoporotic patients.

On the other hand, low BMI and locomotive functions have also been reported as factors that increase the mortality risk [12, 13]. In female osteoporotic patients, low BMI, locomotive functions, and BMD are presumably associated with mortality risk, but factors associated with all-cause mortality have not been identified. Although patients with osteoporosis are expected to have a higher mortality rate, reports focusing exclusively on this population remain limited. Identifying factors contributing to all-cause mortality in female patients with osteoporosis may enable the identification of high-risk patients. An exploratory investigation of factors associated with mortality in osteoporotic patients may contribute to the development of targeted strategies to mitigate mortality risk. We retrospectively studied female patients with osteoporosis living in the community. To the best of our knowledge, this is the first study to quantify mortality specifically in osteoporotic patients and to explore the factors associated with all-cause mortality in this population.

## Materials and methods

Participants

This retrospective cohort study evaluated 451 postmenopausal women who visited the Osteoporosis Outpatient Clinic at Higashi Saitama General Hospital, Satte City, Saitama Prefecture, Japan, between November 2013 and March 2024. Data for research purposes were accessed on March 30 and March 31, 2024. The study included patients who met the diagnostic criteria for osteoporosis of the femoral neck or lumbar spine (T-score ≤ -2.5) and had a follow-up period of at least six months. Exclusion criteria included individuals unable to walk independently, those diagnosed with cancer at baseline, traffic accident, suicide, and patients who withdrew from the study within six months. Radiographic imaging and motor function assessments were conducted at baseline.

Informed consent was obtained from all participants both in writing and verbally. Ethical approval was granted by the ethics committees of Japan University of Health Sciences and Higashi Saitama General Hospital (approval numbers 3001 and 20180005, respectively).

Measurements

Previous studies have reported that mortality is associated with vertebral or femoral fractures, low BMD, BMI, and motor functions [5, 7, 11-13]. This study obtained the information on patient sex, age, underlying diseases (such as hypertension (HT), cardiovascular disease (CD), diabetes mellitus (DM), chronic kidney disease (CKD), and chronic obstructive pulmonary disease (COPD)), history of femoral and vertebral fractures from the medical records at baseline, BMD measurements of the femoral neck and lumbar spine, locomotive syndrome (LS) risk tests, height, and weight as potential predictors of mortality. Additionally, information on incident femoral and vertebral fractures was collected during the follow-up period. These underlying diseases were defined as follows: HT is defined as borderline isolated systolic hypertension (systolic 140-159, diastolic <90 mmHg) or definite isolated hypertension (>160 mmHg); CD is defined as a history of heart failure, acute coronary syndrome, percutaneous coronary intervention, cardiac surgery, or transcatheter aortic valve implantation; DM is defined as fasting plasma glucose ≥126 mg/dl, two-hour oral glucose tolerance test ≥200, or glycated hemoglobin (HbA1C) ≥6.5%; CKD is defined as persistent positive proteinuria and/or estimated glomerular filtration rate (eGFR) < 60 ml/min/1.73 m^2^; and COPD is defined as forced expiratory volume in one second/forced vital capacity ratio <0.70 [14-18]. We measured the BMD of the femoral neck and lumbar vertebrae 1-4 (L1-4), and respective T-scores were calculated by dual-energy X-ray absorptiometry (Horizon, Hologic Japan, Inc., Tokyo, Japan).

Medical information

Previous studies have reported that comorbidities such as HT, DM, CD, CKD, and COPD affect mortality [14-18]. In this study, we measured the presence of comorbidities at baseline, height, and weight as potential predictors affecting mortality. All participants were prescribed medications approved for the treatment of osteoporosis, including bisphosphonates, activated vitamin D, calcitonin, calcium, parathyroid hormone, and denosumab, under the supervision of a physician.

LS risk tests

LS assessments were used in this study to evaluate motor function; the LS assessments include three different measures: the two-step test, the stand-up test, and the geriatric locomotive function scale (GLFS) [19,20].

For the two-step test, participants were instructed to take two maximal strides. The total distance from the starting line to the tip of the toe at the final stopping point was measured. The two-step value was then calculated as the total step length (cm) divided by the participant's height (cm) [19].

For the stand-up test, four platforms of varying heights (10, 20, 30, and 40 cm) were used. Participants were instructed to stand up from a seated position using either one or both legs. The ability to rise unilaterally from the 10, 20, 30, and 40 cm platforms was first assessed. If a participant was unable to stand from the 40 cm platform using one leg, they were instructed to stand using both legs from the 10, 20, 30, and 40 cm platforms. During the one-leg or two-leg stand-up test, participants were required to rise without using momentum (recoil) while keeping their arms crossed and to maintain the standing posture for three seconds. Scoring was based on the highest achieved difficulty level. Points were assigned as follows: eight, seven, six, and five for successfully standing with one leg on the 10, 20, 30, and 40 cm platforms, respectively. If the participant required both legs, scores of four, three, two, and one were assigned for successfully standing from the 10, 20, 30, and 40 cm platforms, respectively. The test was evaluated on an eight-point scale, with the highest level of difficulty scored as eight and the lowest as 0 [19].

For the Geriatric Locomotive Function Scale (GLFS), 25 items were assessed using a five-point scale, with scores ranging from 0 (no disability) to four (severe disability). The total GLFS score was calculated as the sum of all item scores, ranging from a minimum of 0 to a maximum of 100 points. The GLFS comprises four questions related to pain experienced in the past month, 16 questions evaluating activities of daily living (ADL), three questions assessing social functioning, and two questions addressing mental status [20].

The LS risk tests were used to classify the severity of LS into stages based on functional performance and GLFS scores. LS Stage 1 was defined by at least one of the following criteria: a two-step score of <1.3, difficulty standing on one leg from a 40-cm seat (either leg), or a GLFS score >7. LS Stage 2 was defined by at least one of the following criteria: a two-step score of <1.1, difficulty standing with both legs from a 20-cm seat, or a GLFS score >16. LS Stage 3 was defined by at least one of the following criteria: a two-step score of <0.9, difficulty standing with both legs from a 30-cm seat, or a GLFS score >24. [20].

Assessment of mortality

Participants visited the Osteoporosis Outpatient Clinic every six months. If follow-up was not possible, the individual or a relative was contacted to confirm the participant's condition. Confirmation was obtained for deaths at other hospitals, including deaths at our hospital, and information was obtained on the cause and date of death.

Data analysis

The outcome of this study is the incidence of all-cause mortality in females receiving outpatient osteoporosis treatment. In this study, those who survived during the follow-up period (living group) and those who died of all causes for any reason except accidents (deceased group) were included in the analysis.

A Kolmogorov-Smirnov test was conducted to assess the normality of the data. Parametric data following a normal distribution were compared between the living and deceased groups using independent t-tests. For nonparametric data and categorical variables, the Mann-Whitney U test and chi-square test were applied, respectively.

To evaluate the time to mortality incidence during the follow-up period, the Kaplan-Meier method with a log-rank test was used to analyze the severity of LS. Additionally, Cox proportional hazards regression analysis was performed to identify factors associated with mortality during the follow-up period. The follow-up period was defined as the number of years from baseline to the recorded date of death. Covariates included in the Cox proportional hazards regression analysis were age, BMI, history of femoral and vertebral fractures, lumbar spine and femoral BMD, underlying diseases, and LS severity. All statistical analyses were conducted using IBM < SPSS Statistics version 27 for Windows (IBM Corp., Armonk, NY, USA) and EZR (Easy R) version 1.54 for Windows (Saitama Medical Center, Jichi Medical University, Saitama, Japan).

## Results

Incidence of mortality

**Figure 1:**
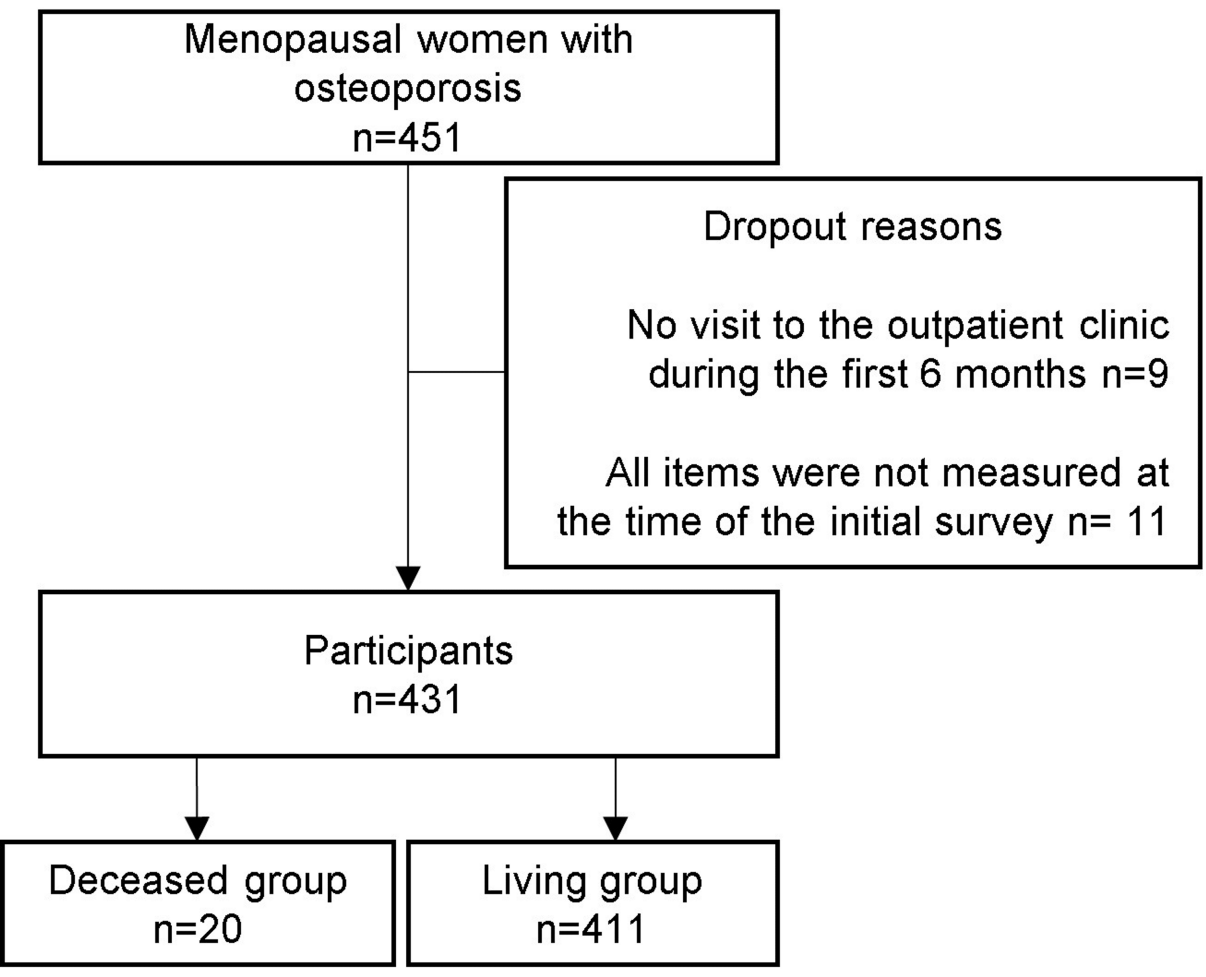
Flowchart showcasing the study population The baseline study included 451 participants; 20 participants dropped out of the study, nine participants dropped out in the first six months, and 11 participants were excluded because they had items that could not be measured during the baseline survey.

Overall, the mean follow-up period was five years (SD: 2.7 years). Nine patients who did not attend the outpatient clinic within the first six months and 11 patients who lacked complete baseline measurements were excluded from the analysis (Figure 1). A total of 20 deaths were recorded, with causes including respiratory failure (n = 3), pneumonia (n = 4), cerebrovascular disease (n = 6), liver cirrhosis (n = 1), heart failure (n = 4), and cancer (n = 2). The mortality incidence rate in this study was 0.91 cases per 100 person-years at risk.

Deceased group vs. living group

This study included 431 patients (95.6%) for follow-up and analysis. The baseline characteristics of the analyzed participants are shown in Table 1. Age, prevalence of hypertension and heart disease, severity of LS, and GLFS were significantly higher in the deceased group than in the living group. Two-step value and stand-up test scores were significantly lower in the deceased group than in the living group. Medication use in the deceased and surviving groups is presented in Table 1, with no significant differences observed between the groups.

**Table 1:** Baseline characteristics of the deceased and living groups P-value, independent samples t-test, Mann–Whitney U test, chi-squared test, and Fisher's exact test. The data for history of femoral and vertebral fractures, hypertension, diabetes mellitus, dyslipidemia, cardiovascular disease, and stroke are presented as the percentage of participants. Height is shown as the mean (standard deviation). Other variables are shown as the median (IQR). BMI: body mass index; BMD: bone mineral density; LS: locomotive syndrome; GLFS: geriatric locomotive function scale; IQR: interquartile range

Parameters		All (n=431)	Deceased group (n=20)	Living group (n=411)	P-value
Age, years		72 (67, 77)	78 (71.5, 79.8)	72 (67, 76)	0.004
Height, cm		150.2 (6.6)	148.8 (9)	150.2 (6.5)	0.345
Weight, kg		50.6 (44.6, 56.5)	49.7 (39.3, 64.1)	50.6 (45, 56.4)	0.823
BMI, kg/m^2^		22.3 (19.9, 25)	22.8 (18.7, 26.9)	22.3 (19.9, 24.9)	0.708
History of femoral fracture, n (%)		17 (3.9)	1 (5.0)	16 (3.9)	0.561
History of vertebral fracture, n (%)		103 (23.9)	6 (30.0)	97 (23.6)	0.512
Hypertension, n (%)		153 (35.5)	12 (60.0)	141 (34.3)	0.019
Cardiovascular disease, n (%)		64 (14.8)	10 (50.0)	54 (13.1)	p<0.001
Diabetes mellitus, n (%)		90 (20.9)	7 (35.0)	83 (20.2)	0.112
Chronic kidney disease, n (%)		29 (6.7)	3 (15.0)	26 (6.3)	0.144
Chronic obstructive pulmonary disease, n (%)		17 (3.9)	1 (5.0)	16 (3.9)	0.804
Rheumatoid arthritis, n (%)		11 (2.6)	0	11 (2.7)	0.589
History of taking glucocorticoids, n (%)		6 (1.4)	0	6 (1.5)	751
BMD L1-L4, g/cm^2^		0.7 (0.7, 0.9)	0.7 (0.6, 0.8)	0.7 (0.7, 0.9)	0.81
T-score L1-L4		-2.3 (-2.9, -1.4)	-2.3 (-3.3, -1.4)	-2.3 (-2.9, -1.4)	0.648
BMD femoral neck, g/cm^2^		0.5 (0.5, 0.6)	0.5 (0.4, 0.6)	0.5 (0.5, 0.6)	0.137
T-score femoral neck		-2.1 (-2.6, -1.6)	-2.2 (-2.9, -1.9)	-2.1 (-2.6, -1.6)	0.142
Bisphosphonates, n (%)		181 (42)	10 (50)	171 (41.6)	0.458
Activated vitamin D, n (%)		224 (52)	9 (45)	215 (52.3)	0.523
Vitamin K2, n (%)		116 (26.9)	6 (30)	110 (26.8)	0.75
Calcium, n (%)		209 (48.5)	13 (65)	196 (47.7)	0.13
Parathyroid hormone, n (%)		54 (12.5)	5 (25)	49 (11.9)	0.084
Denosumab, n (%)		45 (10.4)	3 (15)	42 (10.2)	0.495
Incident femoral fracture, n (%)		25 (5.8)	0	25 (6.1)	0.294
Incident vertebral fracture, n (%)		3 (0.7)	0	3 (0.7)	0.867
Severity of LS		1 (1, 3)	3 (2, 3)	1 (1, 3)	p<0.001
LS Stage 1, n (%)		183 (42.5)	3 (15.0)	180 (43.8)	
LS Stage 2, n (%)		89 (20.6)	6 (30.0)	83 (20.2)	
LS Stage 3, n (%)		115 (26.7)	11 (55.0)	104 (25.3)	
GLFS		10 (4, 23)	21.5 (6, 36.3)	10 (4, 21)	0.018
Stand-up test score		4 (3, 4)	3 (2, 4)	4 (3, 4)	p<0.001
Two-step value, cm/cm		1.21 (1.08, 1.34)	1.02 (0.9, 1.1)	1.22 (1.09, 1.35)	p<0.001

Kaplan-Meier method with the log-rank test

**Figure 2:**
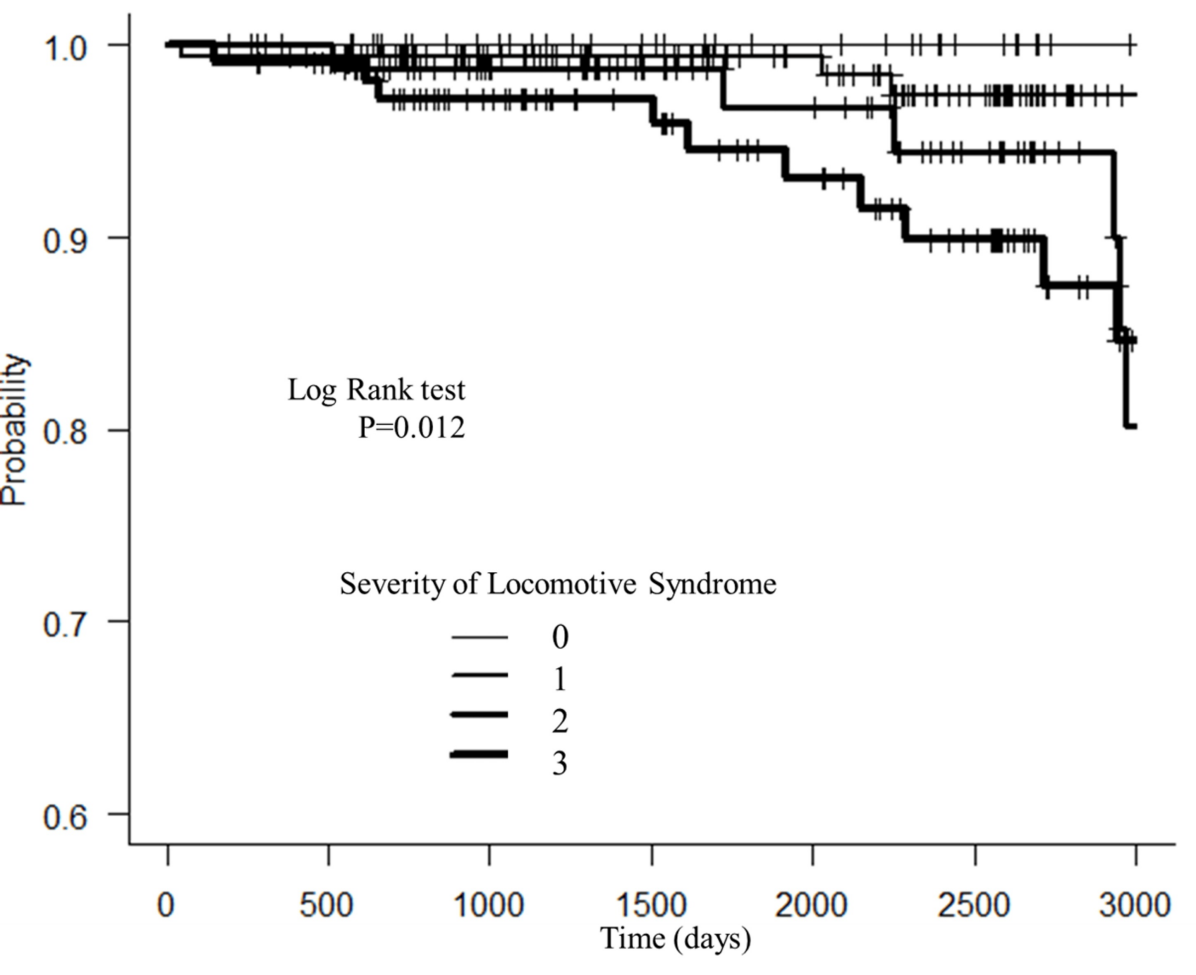
Kaplan–Meier curves Kaplan–Meier curves for the primary endpoint of incidence mortality by severity of locomotive syndrome. A significant difference was observed among the three groups.

Figure 2 shows the data from Kaplan-Meier survival analysis of the primary combined endpoint of incidence of mortality.

The Cox proportional hazards regression analysis

In the Cox proportional hazards regression analysis, the results for the crude ratios, adjusted by age, and adjusted hazard ratios for all measures are shown in Tables 2-4. CD (hazard ratio (HR) = 5.434, 95% confidence interval (CI) 2.256-13.09, p-value < 0.001) and severity of LS (HR = 2, 95% CI 1.159-3.451, 0.013) were independent risk factors for all-cause mortality (Table 3). Additionally, CD (HR = 5.209, 95% CI 1.794-15.128, p-value = 0.002) and LS severity (HR = 2.006, 95% CI 1.063-3.784, 0.032) were independent risk factors for all-cause mortality (Table 4).

**Table 2:** Crude hazard ratios and 95% CI for mortality using the Cox proportional regression analysis 95%CI: 95% confidence interval; BMI: body mass index; BMD: bone mineral density; LS: locomotive syndrome

Parameters	Crude hazard ratio	95%CI	p-value
Age (+1 years)	1.127	1.047 - 1.213	0.001
BMI (+1 kg/m^2^)	1.034	0.923 - 1.159	0.559
History of femoral fracture (+1=presence)	0.73	0.097 - 5.471	0.76
History of vertebral fracture (+1=presence)	1.235	0.478 - 3.189	0.663
Hypertension (+1=presence)	2.328	0.98 - 5.529	0.056
Cardiovascular disease (+1=presence)	3.174	1.346 - 7.486	0.008
Diabetes mellitus (+1=presence)	1.662	0.67 - 4.121	0.273
Chronic kidney disease (+1=presence)	3.47	1.004 - 11.994	0.049
Chronic obstructive pulmonary disease (+1=presence)	1.637	0.218 - 12.289	0.632
Bisphosphonates (+1=presence)	1.199	0.499 - 2.885	0.685
Activated vitamin D (+1=presence)	0.77	0.319 - 1.859	0.562
Vitamin K2 (+1=presence)	0.954	0.365 - 2.491	0.923
Calcium (+1=presence)	1.248	0.488 - 3.19	0.644
Parathyroid hormone (+1=presence)	1.927	0.699 - 5.316	0.205
Denosumab +1=presence)	1.226	0.358 - 4.197	0.746
BMD L1-L4 (+1 g/cm^2^)	0.854	0.039 - 18.794	0.92
BMD femoral neck (+1 g/cm^2^)	0.039	0 - 9.931	0.251
Severity of LS (+1)	2.319	1.372 - 3.918	0.002

**Table 3:** Hazard ratios and 95% CI for mortality using the Cox proportional regression analysis adjusted by age 95%CI: 95% confidence interval; BMI: body mass index; BMD: bone mineral density; LS: locomotive syndrome

Parameters	Adjusted hazard ratio	95%CI	p-value
BMI (+1 kg/m^2^)	1.018	0.905 - 1.145	0.768
History of femoral fracture (+1=presence)	0.546	0.072 - 4.149	0.559
History of vertebral fracture (+1=presence)	1.034	0.395 - 2.708	0.945
Hypertension (+1=presence)	2.27	0.925 - 5.57	0.073
Cardiovascular disease (+1=presence)	5.434	2.256 - 13.09	p<0.001
Diabetes mellitus (+1=presence)	1.61	0.639 - 4.052	0.312
Chronic kidney disease (+1=presence)	2.826	0.816 - 9.782	0.101
Chronic obstructive pulmonary disease (+1=presence)	1.549	0.205 - 11.704	0.671
Bisphosphonates (+1=presence)	1.11	0.461 - 2.672	0.816
Activated vitamin D (+1=presence)	0.829	0.343 - 2.002	0.677
Vitamin K2 (+1=presence)	0.852	0.323 - 2.247	0.746
Calcium (+1=presence)	1.089	0.429 - 2.765	0.858
Parathyroid hormone (+1=presence)	1.86	0.674 - 5.133	0.231
Denosumab +1=presence)	0.947	0.275 - 3.262	0.932
BMD L1-L4 (+1 g/cm2)	0.283	0.011 - 7.163	0.444
BMD femoral neck (+1 g/cm2)	0.222	0.001 - 47.801	0.583
Severity of LS (+1)	2	1.159 - 3.451	0.013

**Table 4:** Hazard ratios and 95% CI for mortality using the Cox proportional regression analysis adjusted by all variable 95%CI: 95% confidence interval; BMI: body mass index; BMD: bone mineral density; LS: locomotive syndrome

Parameters	Adjusted hazard ratio	95%CI	p-value
Age (+1 year)	1.095	0.993 - 1.209	0.07
BMI (+1 kg/m^2^)	0.964	0.83 - 1.119	0.627
History of femoral fracture (+1=presence)	0.653	0.077 - 5.522	0.695
History of vertebral fracture (+1=presence)	0.72	0.212 - 2.449	0.599
Hypertension (+1=presence)	1.538	0.513 - 4.605	0.442
Cardiovascular disease (+1=presence)	5.209	1.794 - 15.128	0.002
Diabetes mellitus (+1=presence)	1.181	0.373 - 3.737	0.777
Chronic kidney disease (+1=presence)	1.303	0.29 - 5.859	0.73
Chronic obstructive pulmonary disease (+1=presence)	1.85	0.219 - 15.63	0.572
Bisphosphonates (+1=presence)	1.416	0.365 - 5.491	0.615
Activated vitamin D (+1=presence)	1.104	0.288 - 4.232	0.885
Vitamin K2 (+1=presence)	0.623	0.185 - 2.095	0.444
Calcium (+1=presence)	1.041	0.343 - 3.163	0.943
Parathyroid hormone (+1=presence)	1.542	0.389 - 6.116	0.538
Denosumab +1=presence)	1.033	0.212 - 5.031	0.968
BMD L1-L4 (+1 g/cm^2^)	0.07	0.001 - 3.381	0.179
BMD femoral neck (+1 g/cm^2^)	1.85	0.083 - 41.478	0.698
Severity of LS (+1)	2.006	1.063 - 3.784	0.032

## Discussion

Among all participants in the study, 20 women died during the follow-up period. Additionally, Cox proportional hazards regression analysis identified age and LS severity as significant predictors of all-cause mortality.

Incidence of all-cause mortality

Jalava et al. reported a mortality rate of 0.9 per 100 per year among older adults without vertebral fractures and 3.16 per 100 per year among the elderly with vertebral fractures [21]. The mortality rate for the study participants was 0.91 deaths per 100 persons per year, suggesting that the mortality rate for the study population was comparable to that of a cohort of elderly persons without vertebral fractures. Furthermore, the majority of deaths were due to respiratory and circulatory diseases. Previous studies have reported that osteoporotic patients are at higher risk of developing cardiac diseases [22]. We believe that many of those who died during follow-up in this study were also at high risk for CD.

Comparison between the mortality group and the living group

Comparing the deceased and living groups, age, prevalence of HT and CD, severity of LS, and GLFS were significantly higher in the deceased group, while two-step value and stand-up test scores were significantly lower in the deceased group. Generally, the factors reported to influence mortality include loss of BMD and fractures [5-7, 11]. On the other hand, there are reports that BMD is not a factor in mortality, and the debate is not unanimous [23]. Almost all previous studies have reported BMD femoral neck as a factor associated with mortality [11] and not with lumbar spine BMD [24]. BMD and history of fractures were not significantly different between the deceased and living groups in this study. We believe that the reason for this result is that all participants in this study had osteoporosis and low bone density, and all were treated for osteoporosis. Additionally, significant differences in locomotive function were observed in the severity of LS, GLFS, the stand-up test score, and the two-step value. The severity of LS indicates a decline in mobility, and each has been reported to be associated with adverse events such as falls and fractures [25]. We believe that age was significantly higher in the deceased group, with a concomitant decline in motor function and higher prevalence of HT and CD. In this study, no incident vertebral fractures or femoral fractures occurred in the deceased group. The mean age of participants in this study was in their 70s, whereas previous studies included a larger proportion of individuals in their 80s [5, 7]. This age difference may explain why fractures were not associated with mortality in the present study.

Predictors of death

In this study, Cox proportional hazards regression analysis was used to explore risk factors associated with mortality. The results showed that the severity of LS was a predictor of death after adjustment for several confounders, including age. Previous studies reported that the two-step test and low gait speed are associated with osteoporosis [26] and a high mortality risk [12], respectively. Additionally, worsening physical function, such as gait speed and standing function, increases the risk of future mortality [27]. Yoshimura et al. [20] reported that each LS risk test value for LS Stage 3 was associated with an increased risk of death. The findings of this study support the previous research by Yoshimura et al. and further emphasize the importance of evaluating LS in patients with osteoporosis by clarifying the causes of death and accurately determining time to death. The LS risk tests are multidimensional measures of mobility function that evaluate vertical mobility with a rise test and horizontal mobility with a two-step test [19]. Although reduced bone density increases the risk of mortality [11], osteoporosis combined with locomotion may further increase the risk of mortality.

In recent years, frailty and reduced bone density and muscle mass (osteosarcopenia) have been associated with a high risk of death. Previous studies indicated that the HR for death from frailty and osteosarcopenia was 1.32-2.63 times higher [28]. In this study, the HR for mortality by LS was 1.98 times higher than that of non-LS, which was comparable to that of frailty and osteosarcopenia. Previous studies have shown that LS occurs in a large proportion of individuals with frailty and sarcopenia [29], indicating a strong association between frailty, sarcopenia, and LS [30]. Although it was impossible to confirm this finding, as frailty and sarcopenia could not be assessed in this study, it is possible that frailty and osteosarcopenia were present among the participants. This study did not compare the severity of LS with that of frailty or osteosarcopenia. However, since the severity of LS has been reported to be the best tool for screening older persons at risk for requiring care in the near future, with a higher rate of those with LS requiring care than those with frailty or sarcopenia [29], we considered that the assessment of the severity of LS could also be useful as a screen for mortality risk in patients with osteoporosis. Interventions for LS, such as locomotion training, in addition to osteoporosis treatment, may be necessary to reduce mortality in patients with both osteoporosis and LS.

There are several limitations to this study. First, this study was a single-site study, which may represent selection bias. As representative mortality rates specifically for osteoporotic patients have not been widely reported, future studies will be essential for contextualizing and comparing the mortality risk of participants in this study. Second, this is a retrospective study using clinical data, and although the relationship between all-cause mortality and the severity of LS was determined, it is possible that not all confounding factors were obtained. In particular, other physical function tests, including gait speed, were not measured in this study, and the effect of the severity of LS on increased mortality and morbidity, including other physical function tests, needs to be clarified in the future. Third, we could only obtain the information from this hospital regarding the patient's medical history, and we did not obtain information about the patient's attendance at other hospitals. These limitations may lead to an overestimation of the importance of physical capacity. Furthermore, the possibility of complications occurring after baseline screening cannot be ruled out. The accurate and precise recording of death, as not all deaths are recorded in the hospital. However, in cases where osteoporosis patients did not attend outpatient visits, clinic staff regularly contacted them by telephone, and the information obtained was documented in their medical records. For deaths that occurred outside the hospital, the causes were also investigated through interviews. We believe this approach provided the most accurate information possible under the circumstances. Fourth, frailty and osteosarcopenia were not directly assessed in this study. Future research should include frailty and osteosarcopenia to determine which is more useful for assessing mortality risk. Finally, the sample size of this study was small, and further research is needed to examine the effects in a fully clinically based population.

## Conclusions

This was an exploratory retrospective cohort study to determine the factors contributing to all-cause mortality in female patients with osteoporosis. The results showed that CD and severity of LS are factors related to all-cause mortality.

To our knowledge, this is the first study to demonstrate an association between the severity of LS and mortality in patients with osteoporosis. We believe that assessing the severity of LS may also serve as a useful screening tool for mortality risk in patients with osteoporosis. Interventions targeting LS, such as locomotion training, in addition to standard osteoporosis treatment, may be necessary to reduce mortality in patients affected by both conditions.
